# The Association Between Puberty Timing and Body Mass Index in a Longitudinal Setting: The Contribution of Genetic Factors

**DOI:** 10.1007/s10519-022-10100-3

**Published:** 2022-04-05

**Authors:** Karri Silventoinen, Aline Jelenkovic, Teemu Palviainen, Leo Dunkel, Jaakko Kaprio

**Affiliations:** 1 Population Research Unit, Faculty of Social Sciences, University of Helsinki, P.O. Box 18, 00014 Helsinki, Finland; 2 Department of Public Health, Faculty of Medicine, University of Helsinki, Helsinki, Finland; 3 Department of Physiology, Faculty of Medicine and Nursing, University of the Basque Country (UPV/EHU), Bilbao, Spain; 4 Institute for Molecular Medicine Finland (FIMM), HiLIFE, University of Helsinki, Helsinki, Finland; 5 Barts & the London Medical School, William Harvey Research Institute, London, UK

**Keywords:** Puberty timing, Body mass index, Genetics, Twins, Polygenic risk scores

## Abstract

We analyzed the contribution of genetic factors on the association between puberty timing and body mass index (BMI) using longitudinal data and two approaches: (i) genetic twin design and (ii) polygenic scores (PGS) of obesity indices. Our data were derived from Finnish cohorts: 9080 twins had information on puberty timing and BMI and 2468 twins also had genetic data. Early puberty timing was moderately associated with higher BMI in childhood in both boys and girls; in adulthood these correlations were weaker and largely disappeared after adjusting for childhood BMI. The largest proportion of these correlations was attributable to genetic factors. The higher PGSs of BMI and waist circumference were associated with earlier timing of puberty in girls, whereas weaker associations were found in boys. Early puberty is not an independent risk factor for adult obesity but rather reflects the association between puberty timing and childhood BMI contributed by genetic predisposition.

**Supplementary Information:**

The online version contains supplementary material available at 10.1007/s10519-022-10100-3.

## Introduction

The timing of puberty is influenced by both genetic and environmental factors. Previous twin studies have shown that from 50% to more than 90% of individual variation of puberty timing is accounted for by inter-individual genetic differences using menarche age in girls (Anderson et al. 2007) and voice break (Hur 2020) and pubertal growth spurt in boys (Silventoinen et al. 2008) as puberty indicators. Further, the role of genetic factors is supported by the associations of parental pubertal timing with different indicators of puberty in offspring (Busch et al. 2020). The importance of genetic factors is also shown in genome-wide-association (GWA) studies on age of menarche in girls (Day et al. 2017; Sarnowski et al. 2021) and voice break in boys (Day et al. 2015). However, there is clear evidence that the timing of puberty has gotten earlier in girls at the same time when general nutrition has improved, showing that factors other than genetic ones are also involved (Papadimitriou 2016; Eckert-Lind et al. 2020). In boys, the evidence is less clear, but a likely reason is that measuring puberty is more difficult in boys than in girls, thus historical data are sparse (Euling et al. 2008).

The secular trend toward the earlier onset of puberty has raised public health concerns because early puberty is associated with an increased risk of metabolic disorders such as type 2 diabetes (Elks et al. 2013) and increased levels of several cardiometabolic risk factors (Widén et al. 2012) as well as an increased breast cancer risk (Collaborative Group on Hormonal Factors in Breast Cancer 2012). However, there is clear evidence that higher childhood body mass index (BMI) and other obesity indices are associated with earlier timing of puberty. The evidence is strongest for girls (Kaplowitz 2008), but there are studies suggesting that high BMI is associated with earlier pubertal timing in boys as well (Busch et al. 2019; Hur 2020; Oehme et al. 2021; Silventoinen et al. 2008). Thus, the associations between puberty timing and cardio-metabolic health in adulthood can also reflect childhood BMI, which is associated with adult BMI leading to metabolic disorders (Lloyd et al. 2012).

Results based on both twin (Silventoinen et al. 2016,2017) and GWA studies (Locke et al. 2015) have shown that genetic factors importantly affect the variation of BMI. The genetic variants associated with BMI are found to be expressed in the brain tissue in particular (Turcot et al. 2018), suggesting the role of behavioral factors as mediators from genes to BMI. Thus, genetic factors can be important when explaining the association between puberty timing and BMI. This is supported by twin studies which have found genetic correlations between BMI and age at voice break (− 0.18) (Hur 2020) and pubertal growth spurt in boys (− 0.16) (Silventoinen et al. 2008). Further, early GWA studies already identified common loci between pubertal timing and BMI in males and females (Cousminer et al. 2013,2014). In more recent studies, the genetic correlation of BMI based on all common genetic variants in the UK Biobank was found to be -0.33 with age at menarche in girls and -0.35 with age at voice break in boys (UK Biobank 2021). A similar genetic correlation between childhood BMI and age at menarche (− 0.42) was found in a large GWA meta-analysis of European-origin populations (Vogelezang et al. 2020).

In this study, we aimed to analyze how the timing of puberty is associated with childhood BMI and BMI in adulthood using longitudinal twin datasets having puberty measures for both males and females. Our specific research aims are (i) to estimate the genetic correlations between puberty indices and BMI measured in adolescence and adulthood using a classical genetic twin design, (ii) to analyze whether the timing of puberty is associated with adult BMI when adjusted for BMI in adolescence, and (iii) to analyze how the genetic liability to obesity measured as polygenic scores (PGS) is associated with the timing of puberty in adolescence. The strength of the data is that the two analytical approaches—the classical twin design utilizing the genetic similarity of twins and the PGS design utilizing measured information on genetic polymorphisms through the whole genome—make different theoretical assumptions thus complementing each other. Briefly, the classical twin design is based on the different genetic relatedness of monozygotic (MZ) and dizygotic (DZ) twins, whereas the PGS design is based on observed associations between genetic polymorphisms and the trait under study (Friedman et al. 2021). Both approaches allows us to calculate shared genetic variation between the timing of puberty and BMI.

## Data and methods

### Study participants

The data were derived from the FinnTwin12 and FinnTwin16 studies, two independent longitudinal cohort studies with the target population of all Finnish twins born in 1983–1987 and 1974–1979, respectively. In the FinnTwin12 study, the baseline questionnaire was sent to 6272 twin individuals at 11–12 years of age and 5184 responded. Three follow-up questionnaires were sent at 14, 17 and, on average 24 years of age (Rose et al. 2019). In the FinnTwin16 study, the baseline questionnaire was sent to 6430 twin individuals at 16 years of age and 5778 responded. Four follow-up questionnaires were sent at 17, 18, 25 and 35 years of age (Kaidesoja et al. 2019). The numbers of valid BMI measures at each age are available in Table 1. In both studies, a sub-cohort of twins were invited to laboratory examinations where they were asked to give a DNA sample (blood, buccal cells or saliva). DNA samples used in the molecular genetic analyses were available for 1438 twins in the FinnTwin12 study and 1030 twins in the FinnTwin16 study. Zygosity was based on measured genotypes for the twins we had a DNA sample. For the rest of the twins, zygosity was based on questions of physical similarity in the baseline questionnaire. This method was validated in the FinnTwin12 study using 395 same-sex twin pairs and in these twins 97% of questionnaire assignment of zygosity was confirmed by a DNA test showing high reliability (Jelenkovic et al. 2011).

**Table 1:** The number of twin individuals and the descriptive statistics of the Pubertal Development Scale (PDS), pubertal age (PA) and body mass index (BMI) by age, sex and study cohort

	Males			Females			p-value of sex difference
	N	Mean	SD	N	Mean	SD	
FinnTwin12 study							
PDS							
12 years	2523	1.30	0.26	2481	1.67	0.44	< 0.0001
14 years	2230	2.02	0.48	2227	2.82	0.53	< 0.0001
BMI (kg/m^2^)							
12 years	2494	17.7	2.55	2416	17.5	2.57	0.036
14 years	2206	19.3	2.69	2267	19.4	2.71	0.884
17 years	2004	21.8	3.02	2154	21.0	2.98	< 0.001
24 years	1417	24.3	3.50	1833	22.8	3.94	< 0.001
FinnTwin16 study							
PA (years)	1704	13.9	1.25	2320	12.8	1.18	< 0.0001
BMI (kg/m^2^)							
16 years	1739	20.4	2.22	2267	20.3	2.43	0.228
17 years	1668	21.1	2.36	2251	20.5	2.45	< 0.0001
18 years	1715	21.8	2.50	2262	20.9	2.67	< 0.0001
25 years	1745	23.8	3.10	2265	22.2	3.39	< 0.0001
35 years	1940	25.7	3.52	2421	23.9	4.38	< 0.0001

### Measures and genotyping

BMI (kg/m^2^) was calculated from self-reported values, which showed high reliability in these cohorts (Saarni et al. 2009). Because of the skewness of BMI distributions at all ages (skewness parameters 1.11–2.36), we log-transformed BMI, producing roughly normal distributions (skewness parameters 0.57–1.08). Sexual maturation in the FinnTwin12 study was measured at 12 and 14 years of age by using the Pubertal Development Scale (PDS), described in detail elsewhere (Dick et al. 2001). The PDS questionnaire includes five items on the timing of secondary sexual characteristics: body hair, growth spurt, skin change, voice change (boys)/breast change (girls), and facial hair (boys)/menarche (girls). The development stage was assessed in a 4-point scale ranging from 1 (no development) to 4 (complete development) except for menarche (1 = has not occurred and 4 = has occurred). At 12 years of age, the fourth response option was not used since only a few children were expected to have complete development. The mean of these ratings was then used as the indicator of puberty; we interpreted that advantaged maturation, i.e., larger PDS scores, indicates the earlier timing of puberty. In the FinnTwin16 study, pubertal age (PA) was asked retrospectively on the age of menarche in girls and voice break in boys at 17 years of age. For those who reported to have not yet experienced menarche/voice break, PA was coded as 17 years of age. BMI and puberty indices were adjusted for age at the time of survey separately in males and females to take into account the age differences between twin pairs within the waves.

The technical details of genotyping, imputation and PGS calculations have been described elsewhere (Kujala et al. 2020). The PGSs were derived using GWA summary statistics of BMI (Yengo et al. 2018), waist-to-hip ratio adjusted for BMI (WHR-BMI) (Pulit et al. 2019) and waist circumference (WC) (Broad Institute 2021). The total number of single nucleotide polymorphisms (SNP) used for the PGS calculations were 996,919 for BMI, 1,148,565 for WHR-BMI and 1,146,408 for WC. The number of individuals in the GWA studies were 692,578 for BMI, 484,563 for WHR-BMI and 419,807 for WC.

### Statistical methods

We started the analyses using classical twin modeling based on the comparisons of similarity between MZ and DZ twins (Posthuma et al. 2003). MZ twins are virtually identical at the gene sequence level, whereas DZ twins share half of genetic variance as in ordinary siblings. Based on this principle, the trait variation can be decomposed to additive genetic variation (A) including all main effects of the loci affecting the trait (correlations of 1.0 within MZ and 0.5 within DZ pairs); dominance genetic variation (D) caused by interactions between alleles in the same locus (correlations of 1.0 within MZ and 0.25 within DZ pairs); shared environment (C) including the effect of all environmental factors that make twins in a pair similar to each other (correlations of 1.0 within both MZ and DZ twins); and unique environment (E) including environmental factors specific to each twin individual as well as any measurement error (correlations of 0 within both MZ and DZ twins). Because we have only twins reared together in our data, we were unable to estimate D and C effects simultaneously. The genetic twin models were fitted using the OpenMx package, version 3.0.2, of R statistical software (Neale et al. 2016).

We estimated the genetic and environmental correlations of PDS with BMI at different ages by using bivariate Cholesky decomposition, a method decomposing all variance and covariance in the data into uncorrelated factors. Further, we estimated these correlations between PDS at 12 and 14 years of age to analyze how much they reflect the same genetic factors. Thus, separate bivariate models were conducted at each age. Since puberty is later in boys than in girls and thus the puberty indices are not necessarily comparable between sexes, we used only same-sex pairs in these analyses. The number of complete pairs (MZ and same-sex DZ) in each bivariate model varied between 822 and 1475 for PDS (FinnTwin12 study) and between 930 and 978 for PA (FinnTwin16 study) (Supplementary Table 1). We then analyzed the associations of PDS and PA with BMI in adulthood after adjusting for the baseline BMI using the linear regression model. If independent associations between the puberty indices and post-puberty BMI existed, we decomposed the regression residuals to additive genetic and unique environmental components.

The heritability estimates of puberty indices and BMI have previously been reported in these study cohorts, suggesting the presence of dominance genetic factors for PA (Kaprio et al. 1995) and shared environment for PDS in females (Wehkalampi et al. 2008) and shared environment for BMI until 14 years of age in both males and females (Lajunen et al. 2009). At later ages, an additive genetic/unique environment (AE) model was the best fitting model for BMI (Lajunen et al. 2009; Ortega-Alonso et al. 2012). Thus, we selected the additive genetic/shared environment/unique environment (ACE) model as the starting point of genetic modeling. The model fit statistics are presented in Supplementary Table 1. We did not find evidence that there would be shared environmental correlations affecting the associations between PDS or PA and BMI. This correlation was only statistically significant at conventional level in the model for PDS and BMI at 12 years of age in males (p = 0.0171). However, this may be a spurious finding because of multiple testing. When we compared the AE models to the ACE models, for 6 models we found a statistically significant difference at the conventional level (p = 0.004–0.0400), but they were not statistically significant after the Bonferroni correction of multiple testing (significance level p = 0.002 for 24 tests). We decided to use the AE model in the statistical analyses; if the shared environmental variance component is estimated without simultaneously estimating the shared environmental correlation, it would have artificially increased the additive genetic correlation.

After these genetic twin models, we continued the analyses by studying how the PGSs of obesity indices were associated with the puberty indices. In these analyses, we used linear regression models with puberty indices as the dependent and PGS as an independent variable. The population stratification was adjusted for by including ten principal components of the genome into the model as independent variables. We calculated standardized β-coefficients presenting the change of 1 standard deviation (SD) of PDS or PA per the change of 1 SD of each PGS of obesity indices. The PGS analyses, as well as all statistical testing in the descriptive analyses, were performed using linear regression models by the Stata/SE 16.1 for Windows statistical software (StataCorp, College Station, TX, USA). The cluster option was used to correct the standard errors and confidence intervals (CI) for the lack of statistical independence of twins sampled as twin pairs (Williams 2000).

## Results

Table 1 presents the descriptive statistics of our study cohort. Boys developed later than girls in their sexual maturation measured by PDS at 12 and 14 years of age or retrospectively asked PA at 17 years of age. Boys had slightly higher BMI than girls at 12 years of age. This difference disappeared by 14 years of age but emerged again at 17 years of age. The sex difference in BMI increased during adulthood and was largest at 35 years of age when men had 1.79 (95% CI 1.52–2.05) kg/m^2^ higher BMI than women.

We started the analyses by studying the contributions of genetic and environmental factors on the correlations between PDS, PA and BMI at different ages (Table 2). Separate bivariate Cholesky decomposition was used at each age to decompose the trait correlation into additive genetic correlation and unique environmental correlation. We also calculated the proportions of the trait correlations explained by additive genetic and unique environmental factors. A modest correlation was observed between PDSs at ages 12 and 14 (r = 0.36 in boys and 0.51 in girls), which was nearly totally explained by genetic factors: the genetic correlations showed that 24% of genetic variation in boys and 38% in girls affecting sexual maturation was shared between these ages. BMI was positively correlated with PDS at both ages: the correlations varied from 0.14 in boys at 12 years of age to 0.28 in girls at 14 years of age. Very similar correlations (-0.15 in boys and -0.28 in girls) were also found when studying PA and BMI at the age of 16. For the most part (84% or more), these correlations were explained by genetic factors. The genetic correlations showed that from 3 to 11% of genetic variation of adolescence BMI and puberty indices was explained by the same genetic factors. When we studied the correlations between the puberty indices and post-puberty BMI, they systematically decreased over age. However, these correlations were again mainly explained by shared genetic factors (64% or more).

**Table 2:** The trait correlations of the Pubertal Development Scale (PDS), pubertal age (PA) and body mass index (BMI) between different ages and the correlations between additive genetic and unique environmental variance components explaining these trait correlations by sex

Trait 1	Trait2	Trait correlations			Additive genetic correlations				Unique environmental correlations			
		r	95% confidence intervals		r_A_	95% confidence intervals		% of r explained by r_A_^1^	r_E_	95% confidence intervals		% of r explained by r_E_^a^
			LL	UL		LL	UL			LL	UL	
Males												
PDS12	PDS14	0.36	0.31	0.41	0.49	0.41	0.56	96	0.05	− 0.05	0.16	4
PDS12	BMI12	0.14	0.08	0.19	0.17	0.09	0.24	95	0.03	− 0.07	0.12	5
PDS14	BMI14	0.17	0.11	0.22	0.21	0.13	0.29	96	0.03	− 0.08	0.14	4
PDS12	BMI17	0.09	0.03	0.15	0.09	0.00	0.17	75	0.10	− 0.01	0.21	25
PDS12	BMI22	0.10	0.03	0.17	0.09	− 0.02	0.20	64	0.12	0.00	0.23	30
PDS14	BMI17	0.13	0.07	0.20	0.19	0.10	0.27	106	− 0.04	− 0.16	0.08	− 6
PDS14	BMI24	0.12	0.05	0.20	0.15	0.04	0.27	87	0.05	− 0.09	0.19	12
PA	BMI16	− 0.15	− 0.23	− 0.08	− 0.23	− 0.33	− 0.12	109	0.06	− 0.09	0.20	− 9
PA	BMI17	− 0.11	− 0.19	− 0.04	− 0.12	− 0.23	− 0.02	86	− 0.08	− 0.23	0.08	14
PA	BMI18	− 0.08	− 0.16	0.00	− 0.09	− 0.20	0.03	77	− 0.07	− 0.21	0.07	23
PA	BMI25	− 0.06	− 0.14	0.02	− 0.06	− 0.17	0.07	68	− 0.07	− 0.20	0.07	32
PA	BMI35	− 0.06	− 0.14	0.02	− 0.06	− 0.19	0.06	76	− 0.05	− 0.19	0.09	24
Females												
PDS12	PDS14	0.51	0.47	0.55	0.62	0.56	0.68	93	0.15	0.05	0.25	7
PDS12	BMI12	0.24	0.19	0.30	0.25	0.18	0.32	84	0.21	0.11	0.30	16
PDS14	BMI14	0.28	0.22	0.33	0.33	0.26	0.41	90	0.12	0.02	0.22	10
PDS12	BMI17	0.13	0.07	0.19	0.17	0.09	0.25	95	0.03	− 0.08	0.13	5
PDS12	BMI22	0.13	0.06	0.19	0.15	0.07	0.24	93	0.04	− 0.08	0.15	7
PDS14	BMI17	0.14	0.08	0.20	0.18	0.09	0.27	89	0.05	− 0.05	0.15	11
PDS14	BMI24	0.13	0.07	0.20	0.20	0.10	0.29	107	− 0.03	− 0.14	0.08	− 7
PA	BMI16	− 0.28	− 0.34	− 0.22	− 0.30	− 0.38	− 0.23	90	− 0.18	− 0.28	− 0.08	10
PA	BMI17	− 0.22	− 0.28	− 0.16	− 0.25	− 0.33	− 0.18	96	− 0.06	− 0.17	0.05	4
PA	BMI18	-0.19	− 0.25	− 0.13	− 0.21	− 0.29	− 0.13	92	− 0.09	− 0.19	0.02	8
PA	BMI25	− 0.18	− 0.24	-0.11	− 0.20	− 0.29	− 0.12	90	− 0.08	− 0.19	0.03	10
PA	BMI35	− 0.17	− 0.23	− 0.11	− 0.17	− 0.25	− 0.08	77	− 0.19	− 0.29	− 0.08	23

^a^The proportion (%) of trait correlation explained by additive genetic (r_A_) and unique environmental co-variation (r_E_) shared by trait 1 and trait 2. The negative values indicate that the co-variation affects in the opposite direction than the trait correlation

Next, we analyzed how the puberty indices were associated with post-pubertal BMI after adjusting the results for baseline BMI (Table 3). However, only weak and mainly statistically non-significant associations were found. The only statistically significant associations were found between PA and BMI at 17 and 18 years of age in females, but these associations were also small in size (0.03 and 0.06 SD of BMI per 1 SD change in PA) and faded with later age. Thus, we did not further decompose these associations into genetic and environmental factors.

**Table 3:** The standardized regression coefficients of the Pubertal Development Scale (PDA) and pubertal age (PA) for post puberty body mass index (BMI) by age and sex

Age	Males			Females		
	β	95% confidence intervals		β	95% confidence intervals	
		LL	UL		LL	UL
PDS12^a^						
BMI17	0.03	− 0.01	0.06	− 0.03	− 0.06	0.01
BMI24	0.03	− 0.02	0.08	− 0.04	− 0.08	0.01
PDS14^b^						
BMI17	0.00	− 0.03	0.04	− 0.07	− 0.11	− 0.03
BMI24	0.01	− 0.04	0.07	− 0.04	− 0.08	0.00
PA^c^						
BMI17	− 0.01	− 0.04	0.03	0.03	0.01	0.06
BMI18	0.02	− 0.02	0.06	0.06	0.03	0.09
BMI25	0.03	− 0.02	0.07	0.02	− 0.01	0.06
BMI35	0.01	− 0.04	0.05	− 0.02	− 0.06	0.02

^a^Adjusted for BMI at 12 years of age

^b^Adjusted for BMI at 14 years of age

^c^Adjusted for BMI at 16 years of age

Finally we analyzed how the PGSs of different obesity indices were associated with puberty timing (Table 4). In girls, the high PGSs of both BMI and WC were associated with more advanced maturation measured by PDS both at age 12 and 14. However, at 12 years of age, this association was only marginally statistically significant for the PGS of WC. The strongest association was found at 14 years of age when a 1 SD increase of the PGS of WC increased the PDS by 0.13 SD. In boys, similar associations were found, but they were weaker than in girls and not statistically significant. When using retrospectively reported PA, the associations were weak and not statistically significant. We did not find systematic associations between the PGS of WHR-BMI and the puberty indices.

**Table 4:** The standardized regression coefficients of polygenic scores of obesity indices for the Pubertal Development Scale (PDS) and pubertal age (PA) by sex

Polygenic scores	Males				Females			
	N	β	95% confidence intervals		N	β	95% confidence intervals	
			LL	UL			LL	UL
PDS at 12 years of age								
Body mass index	636	0.03	− 0.05	0.11	781	0.08	0.01	0.16
Waist circumference	636	0.05	− 0.03	0.14	781	0.07	− 0.01	0.15
Waist-to-hip ratio adjusted for body mass index	636	0.00	− 0.09	0.09	781	0.00	− 0.08	0.08
PDS at 14 years of age								
Body mass index	605	0.07	− 0.01	0.15	756	0.12	0.04	0.20
Waist circumference	605	0.06	− 0.02	0.15	756	0.13	0.05	0.21
Waist-to-hip ratio adjusted for body mass index	605	− 0.08	− 0.17	0.01	756	− 0.02	− 0.09	0.06
Pubertal age								
Body mass index	436	0.03	− 0.10	0.15	594	− 0.09	− 0.19	0.02
Waist circumference	436	0.03	− 0.11	0.16	594	0.02	− 0.08	0.12
Waist-to-hip ratio adjusted for body mass index	436	− 0.04	− 0.15	0.07	594	0.04	− 0.07	0.14

^a^Adjusted for 10 principal components of genetic population stratification. The change of 1 standard deviation of PDS per the change of 1 standard deviation of puberty indices

## Discussion

In our study based on two independent longitudinal cohorts of twins, we found clear evidence that there is shared genetic overlap between the timing of puberty and BMI being the strongest in childhood but still existing in adulthood. When using classical twin modeling, the results were very systematic for males and females: the genetic correlations were moderate between puberty timing and childhood BMI and decreased when studying BMI in adulthood. When studying the associations between the PGSs of adiposity indices and puberty timing, the associations were stronger in females than in males. However, the confidence intervals were also wide and the results were not statistically significant for males. Thus, larger data sets are needed to analyze the question of sex differences in detail. However, in general, our results correspond well with previous twin studies (Hur 2020; Silventoinen et al. 2008) and GWA studies (Cousminer et al. 2013; Cousminer et al. 2014; UK Biobank 2021; Vogelezang et al. 2020) that also show shared genetic variation between the timing of puberty and BMI. Further, previous studies have also shown that the PGSs of puberty indices are associated with BMI in childhood and adulthood, providing further evidence on the shared genetic background (Bell et al. 2018; Gill et al. 2018). Our results confirm that a large proportion, more than 90% at most ages, of the correlations between the timing of puberty and BMI is due to common genetic factors. Together, these results provide strong evidence that the same genetic factors affecting early puberty and childhood adiposity explain the association between the timing of puberty and BMI in adulthood. However, it is noteworthy that at maximum only 11% of the genetic variation is shared between puberty timing and adolescence BMI, thus most of the genetic variation is specific for these two traits.

When studying the longitudinal associations, we found only a little evidence that the timing of puberty is associated with adult BMI independently of pre-pubertal BMI. We found that the early timing of puberty was associated in males and females with higher adult BMI, but the correlations were weaker than with childhood BMI and largely disappeared when adjusted for childhood BMI. These results are largely similar to those found previously in population-level studies, but it is noteworthy that only a few studies so far have studied these association in males (Prentice and Viner 2013). The conclusion that pre-pubertal fat mass in particular is behind the association between the earlier timing of puberty and later adiposity in males and females is supported by a UK study having longitudinal data of growth and direct measures of fat mass based on dual-energy X-ray absorptiometry (DEXA) scans from 9 to 18 years (O'Keeffe et al. 2020).

Our findings are consistent with Mendelian randomization analyses from the UK Biobank finding that high early BMI was associated with earlier timing of puberty whereas adult BMI has a negligible effect after controlling for early BMI (Richardson et al. 2020). This same study also found that prepubertal BMI and the timing of puberty act through adult BMI to affect the risk of coronary heart disease and type 2 diabetes. However, for breast cancer the associations were different and the study showed strong evidence for a direct causal effect of earlier age of menarche on higher risk of breast cancer independent of early life or adult body size (Richardson et al 2020). The evidence of a causal effect of early puberty on breast cancer risk was also found in twin studies where earlier puberty was associated with earlier diagnosis of breast cancer in MZ twin pairs concordant for breast cancer (Hamilton & Mack 2003) and with higher breast cancer risk in twin pairs discordant for early-onset breast cancer (Swerdlow et al 2002). This evidence suggests that even when early puberty is associated with many diseases, the causal pathways between early BMI, puberty timing, adult BMI and disease risk can vary according to the specific disease.

The mechanisms behind the found associations are still unclear. The shared genetic background of the timing of puberty and obesity can be due to a pleiotropic effect but also causality. If higher childhood adiposity affects the earlier timing of puberty, it can create a genetic correlation between these traits. Further, our results do not exclude the possibility that environmental factors can contribute to this association. BMI has globally increased during the recent decades (NCD Risk Factor Collaboration 2016), but the heritability estimates of BMI have been largely constant since both genetic and environmental variations have increased at the same time (Silventoinen et al. 2017). In a similar way, environmental factors may affect more children having genetic susceptibility to adiposity, and increased fat mass can then affect the earlier timing of puberty. Regardless of the mechanisms behind this association, the genetic background between the timing of puberty and BMI supports the conclusion that early puberty is not an independent risk factor of adult obesity, but rather that the origin of this association is in childhood.

Our data have both strengths and weaknesses. Our main strength is that we have longitudinal information on puberty timing and BMI in genetically informative data in both males and females. Having puberty measures for males is a major advantage, since most of the previous studies have used menarche age as a puberty indicator and thus include only females. Additionally, BMI and the timing of puberty were measured in adolescence thus decreasing recall bias which is a problem especially in males. However, it is noteworthy that PA was measured retrospectively at age 17 and was only based on one question, which may have increased measurement error. This may explain why our results for PA were generally weaker than for PDS. We were able to use two genetic designs—classical genetic twin study and molecular genetic data—each making different theoretical assumptions and thus increasing the robustness of the results. Our main weakness is that we needed to rely on self-reported data on measuring both puberty and obesity. Having the assessment of puberty stage done by trained healthcare professionals and measures of fat mass by magnetic resonance imaging or DEXA would have increased the preciseness of our results. Further, our study size was somewhat underpowered for the analyses using the PGSs of obesity indices, especially when testing sex differences.

In conclusion, our results indicate that early puberty is not an independent risk factor of adult obesity but rather reflects the genetic predisposition to obesity. Creating less obesogenic environments for children would be especially important for those children having genetic susceptibility to adiposity and thus a higher risk for early puberty.

## Supplementary Information

Below is the link to the electronic supplementary material.

Supplementary file1 (DOCX 35 kb)

## Data Availability

Because of the consent given by study participants and the high degree of identifability, data cannot be made publicly available. Data are available through the Institute for Molecular Medicine Finland (FIMM) Data Access Committee (DAC) for authorized researchers who have IRB/ethics approval and an institutionally approved study plan. For more details, please contact the FIMM DAC (fimm‐dac@helsinki.f).

## Code Availability

Computer codes are available from the corresponding author.
