# Supplementary material for: The Association Between Puberty Timing and Body Mass Index in a Longitudinal Setting: The Contribution of Genetic Factors
Source: Behav Genet. 2022 Apr 5;52(3):186–94. doi: 10.1007/s10519-022-10100-3 (PMC9135891; doi:10.1007/s10519-022-10100-3)
Supplement: Supplementary file 1 — Supplementary file1 (DOCX 35 kb) [file 10519_2022_10100_MOESM1_ESM.docx]

Supplementary table 1. Number of complete twin pairs used in analyzes and model fit statistics for bivariate Cholesky decompositions by sex.

| **Trait 1** | **Trait2** | **N of complete twin pairs** | | **Full ACE model** | | **ACE model without C correlation^1^** | | **AE model^2^** | |
| --- | --- | --- | --- | --- | --- | --- | --- | --- | --- |
|  |  | **MZ** | **DZ** | **d.f.** | **-2LL** | **Δ -2LL** | **p-value** | **Δ -2LL** | **p-value** |
| **Males** | |  |  |  |  |  |  |  |  |
| PDS12 | PDS14 | 326 | 342 | 3033 | 7929.16 | 0.08 | 0.7733 | 6.17 | 0.1038 |
| PDS12 | BMI12 | 369 | 395 | 3204 | 1778.24 | 5.68 | 0.0171 | 18.35 | 0.0004 |
| PDS14 | BMI14 | 315 | 335 | 2831 | 1574.03 | 0.06 | 0.8113 | 4.36 | 0.2249 |
| PDS12 | BMI17 | 294 | 306 | 2895 | 2211.76 | 1.68 | 0.1948 | 8.31 | 0.0400 |
| PDS12 | BMI22 | 195 | 170 | 2508 | 3053.72 | 0.00 | 0.9563 | 6.03 | 0.1101 |
| PDS14 | BMI17 | 285 | 294 | 2711 | 1740.75 | 0.00 | 1.0000 | 0.00 | 1.0000 |
| PDS14 | BMI22 | 183 | 159 | 2324 | 2586.49 | 0.09 | 0.7680 | 0.52 | 0.9145 |
| PA | BMI16 | 170 | 189 | 1641 | 534.55 | 2.04 | 0.1537 | 2.25 | 0.5219 |
| PA | BMI17 | 165 | 186 | 1617 | 542.89 | 1.71 | 0.1915 | 1.76 | 0.6241 |
| PA | BMI18 | 172 | 199 | 1644 | 723.42 | 2.10 | 0.1469 | 2.18 | 0.5364 |
| PA | BMI25 | 170 | 182 | 1646 | 961.20 | 0.77 | 0.3817 | 0.77 | 0.8572 |
| PA | BMI35 | 174 | 198 | 1663 | 973.35 | 0.01 | 0.9403 | 0.02 | 0.9992 |
| **Females** | |  |  |  |  |  |  |  |  |
| PDS12 | PDS14 | 358 | 304 | 2962 | 7161.45 | 3.30 | 0.0693 | 7.41 | 0.0598 |
| PDS12 | BMI12 | 382 | 329 | 3056 | 1470.72 | 1.17 | 0.2794 | 10.16 | 0.0172 |
| PDS14 | BMI14 | 353 | 304 | 2836 | 1314.34 | 0.11 | 0.7436 | 3.99 | 0.2623 |
| PDS12 | BMI17 | 346 | 301 | 2931 | 1739.44 | 0.03 | 0.8648 | 7.79 | 0.0506 |
| PDS12 | BMI22 | 285 | 233 | 2720 | 2432.24 | 0.00 | 0.9643 | 10.38 | 0.0156 |
| PDS14 | BMI17 | 337 | 289 | 2792 | 1640.87 | 0.20 | 0.6523 | 0.50 | 0.9196 |
| PDS14 | BMI22 | 265 | 215 | 2581 | 2334.50 | 0.00 | 0.9643 | 2.51 | 0.4729 |
| PA | BMI16 | 335 | 271 | 2522 | 724.43 | 0 | 1 | 0 | 1 |
| PA | BMI17 | 333 | 271 | 2515 | 768.98 | 0 | 1 | 0 | 1 |
| PA | BMI18 | 339 | 268 | 2521 | 998.08 | 0 | 1 | 0 | 1 |
| PA | BMI25 | 319 | 259 | 2497 | 1531.29 | 0 | 1 | 0 | 1 |
| PA | BMI35 | 333 | 266 | 2503 | 1935.52 | 0 | 1 | 0 | 1 |

^1^Compared to full ACE model (Δ 1 d.f.); ^2^Compared to full ACE model (Δ 3 d.f.);
